# Epithelioid Rhabdomyosarcoma; a case report with immunohistochemical and molecular study

**DOI:** 10.1186/s13000-015-0349-2

**Published:** 2015-07-25

**Authors:** Ryu Jokoji, Jun-ichiro Ikeda, Masahiko Tsujimoto, Eiichi Morii

**Affiliations:** Department of Pathology, Nissay Hospital, 6-3-8 Itachibori, Nishi-ku, Osaka, 550-0012 Japan; Department of Pathology, Osaka Police Hospital, Osaka, Japan; Department of Pathology, Osaka University Graduate School of Medicine, Osaka, Japan

**Keywords:** Rhabdomyosarcoma, Adult, RT-PCR, Immunohistochemistry, Karyotype

## Abstract

Recently, we encountered a biopsy of epithelioid rabdomyosarcoma with lymph node metastasis. A computed tomography (CT) scan showed number of swollen lymph nodes in the left neck and a huge abdominal mass occupying the right kidney. In the lymph node biopsy, tumor cells showed diffuse sheet-like growth reminiscent of carcinoma and melanoma cells with extensive distribution of coagulation necrosis. Tumor cells had abundant amphophilic cytoplasm and clear large nuclei. Most tumor cells showed severe cytologic atypia manifested in prominent nucleoli and pleomorphic nuclei. Tumor cells were focally positive for desmin. Most tumor cells showed expressons for vimentin, BAF47 (INI-1), and myogenin. On reverse transcriptase polymerase chain reaction (RT-PCR) analysis, tumor cells lacked Myo D1 and PAX3/7-FKHR transcripts and showed myogenin transcripts. On cytogenetic (karyotypic) analysis, tumor cells showed highly complex karyotypes. The patient received various regimens of chemotherapy, but 6 months after the biopsy she died with progression of the tumor. Since consent was not obtained, an autopsy was not performed.

**Electronic supplementary material:**

The online version of this article (doi:10.1186/s13000-015-0349-2) contains supplementary material, which is available to authorized users.

## Background

Rhabdomyosarcoma(RMS) is classified by the current World Health Organization (WHO) into four major subtypes, embryonal RMS (ERMS), alveolar RMS (ARMS), pleomorphic RMS (PRMS), and spindle cell/sclerosing RMS (SRMS) [1]. Recently, a part of RMS demonstrated epithelioid morphorogy reminiscent of poorly differentiated carcinoma or melanoma and caused difficulty in diagnosis. Previous reports had identified these cases as epithelioid RMS (epiRMS) [2].

**Fig. 1:**
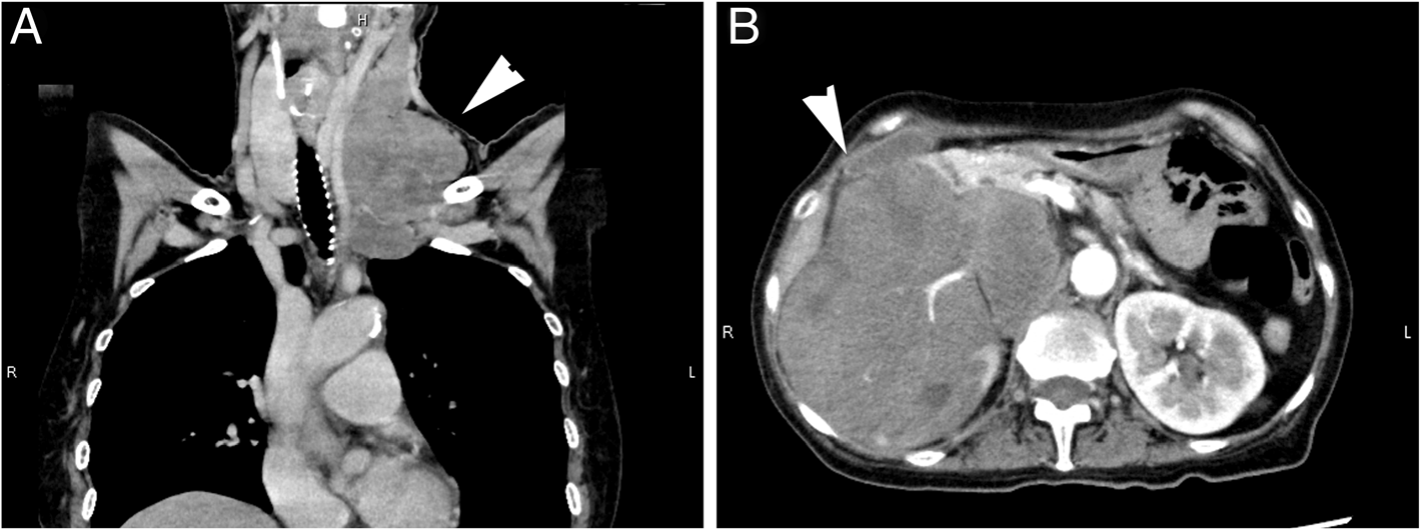
Clinical images: Computed tomography (CT), obtained before the biopsy, showing the swollen lymph nodes in the left neck (**a**, *white arrow head*) and a huge abdominal mass occupying the right kidney (**b**, *white arrow head*) as low-density masses

**Fig. 2:**
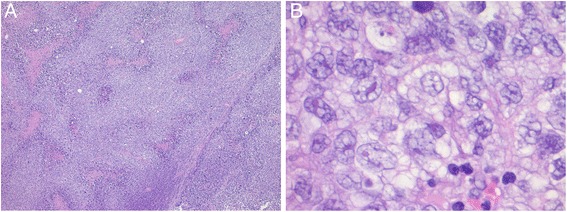
Microscopic images: (**a**) Tumor cells showing diffuse sheet-like growth with extensive distribution of coagulation necrosis. **b** Tumor cells with abundant amphophilic cytoplasm and clear large nucleus with severe cytological atypia in the form of prominent nucleoli and pleomorphic nuclei

**Fig. 3:**
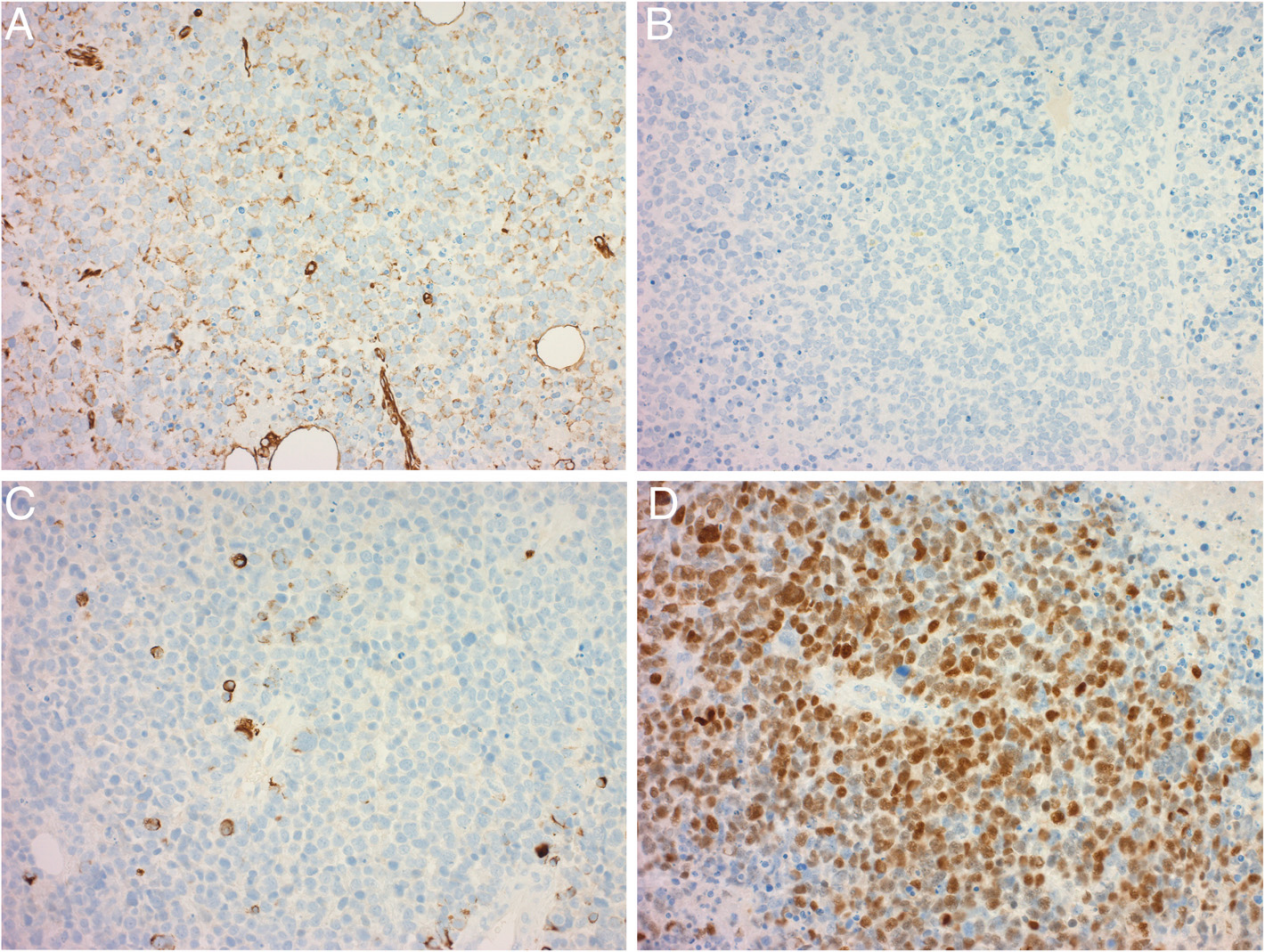
Immunohistochemical images: Tumor cells stained weakly positive for vimentin (**a**) and negative for cytokeratin (**b**). Tumor cells stained focally positive for desmin (**c**) and diffusely positive for myogenin (**d**)

We encountered a case of epiRMS with nodal metastasis, for which an extensive immunohistochemical and molecular study was performed.

## Case presentation

A 65-year-old female patient visited our clinic, complaining of low back pain, general fatigue and cervical masses. Computed tomography (CT) detected number of swollen lymph nodes in the left neck and a huge abdominal mass occupying the right kidney. Tumor growth had spread to retroperitoneal, regional and para aortic lymph nodes, and the aorta. CT showed no finding that tumor had been originated from a large nerve (Fig. 1). There was no significant difference in the CT value between abdominal primary tumor and metastatic cervical lymph nodes (70–90 Huns Hounsfield Unit (HU) and 60–90 HU). Both lesions were suggested to be constructed from substantially the same components. For histological diagnosis, cervical lymph node biopsy was performed.

Microscopically, tumor cells showed diffuse sheet-like growth reminiscent of carcinoma and melanoma cells with extensive distribution of coagulation necrosis. Tumor cells had abundant amphophilic cytoplasm and a clear large nucleus. Most tumor cells showed severe cytologic atypia manifested in the form of prominent nucleoli and pleomorphic nuclei. Tumor cells with bizarre nucleus were not found. No cross striations were observed (Fig. 2). 

Immunohistochemistry for cytokeratin, LCA, S-100, Sox10, Melan A, smooth muscle actin, h-Caldesmon, MDM2, CDK4, p16 and Myo D1 was negative for all tumor cells. Tumor cells were focally positive for desmin. Most tumor cell showed weak expression for vimentin and diffuse expression for BAF47(INI-1), and myogenin (Fig. 3). 

On reverse transcriptase polymerase chain reaction (RT-PCR) analysis, tumor cells lacked Myo D1, PAX3/7-FKHR transcripts and showed myogenin transcripts. On cytogenetic (karyotypic) analysis, tumor cells showed highly complex karyotypes with triploidy and structural rearrangements (Additional file 1: Figures S1-3 and Tables S1-3).

The final diagnosis was metastatic rhabdomyosarcoma with epithelioid morphology that originated from the right kidney or retroperitoneum. From morphological, immunohistochemical, cytogenetical and molecular analyses, we diagnosed the tumor to be a epiRMS. The patient received various regimen of chemotherapy, but 6 months after the biopsy she died with progression of the tumor. Since consent was not obtained, an autopsy was not performed.

## Conclusion

Epithelioid RMS was recently reported as a distinct morphological variant of RMS.

RMS is classified by the current WHO into four major subtypes, ERMS, ARMS, PRMS, and SRMS. In previous reports with regard to other types of RMS, ERMS was characterized by primitive mesenchymal cells showing various stage of myogenesis and exhibited complex karyotypes with numerical and structural rearrangements, including polysomies of chromosomes 2, 8, 11, 12, and 13 [3, 4]. ARMS was typically characterized by primitive round cells surrounded by fibrovascular stroma and exhibited recurrent translocations, t(2; 13)(q35; q14)(PAX3-FKHR) and t(1; 13)(q35; q14)(PAX7-FKHR) in approximately 85% of cases. PAX3/7-FKHR fusion is specific to ARMS [5, 6]. PRMS was characterized by nuclear pleomorphism and bizarre polygonal eosinophilic cells and exhibited an extremely complex karyotype with numeric and structural rearrangements without specific genetic abnormality [7, 8]. SRMS was characterized by spindle cells and various degree of stromal hyalinization and exhibited aneuploidy without specific genetic abnormality in only few reports [9–12].

Histologically, epiRMS showed diffuse sheet-like growth of uniformly sized epithelioid cells with abundant amphophilic to eosinophilic cytoplasm, large vesicular nuclei, and frequent prominent nucleoli, reminiscent of poorly differentiated carcinoma or melanoma. Consequently, its morphology caused difficulty in diagnosis [2]. Tumor cells showed skeletal muscle differentiation on immunohistochemical analysis, such as Myo D1 and/or myogenin. One of the differential diagnoses includes PRMS. However epiRMS lacks the obvious nuclear pleomorphism and bizarre polygonal eosinophilic cells that are characteristic of PRMS. Although most reports of epiRMS were of late elderly onset in the elderly, cases in children and young people have also been reported [2, 13–18].

In our case, the diagnosis of epiRMS was extremely difficult. The differential diagnosis for epiRMS includes poorly differentiated carcinoma, malignant melanoma, and epithelioid sarcoma. Morphologically, diffuse sheet-like growth pattern and severe cytologic atypia in the form of prominent nucleoli initially suggested carcinoma and melanoma cells. However, this was dismissed by immunohistochemical analysis that showed negative staining for cytokeratin, Melan A and S-100. Diffuse sheet-like epithelioid growth pattern with extensive distribution of necrosis and positive staining for vimentin suggested epithelioid sarcoma. However, this was rejected because immunohistochemistry showed positive staining for BAF47 (INI-1). In our case, since biopsy specimen only was evaluated, it might be part of tumor with rahbdomyosarcoma component, for example, dedifferentiated liposarcoma, Triton tumor, Rhabdoid tumor and carcinosarcoma. The differential diagnosis for dedifferentiated liposarcoma and Rhabdoid tumor were dismissed by immunohistochemical analysis that showed negative staining for MDM2, CDK4, p16 and positive staining for BAF47(INI1). The differential diagnosis for Triton tumor was dismissed by immunohistochemical analysis that showed negative staining for Sox10, S-100 and findings of abdominal CT. PRMS-like morphology, a sheets of large and atypical polygonal eosinophilic cells or of undifferentiated round to spindle cells with various degree of cross-striation, is more seen as a heterologous component in carcinosarcoma and dedeifferentiated liposarcoma, among others [1]. Furthermore from findings of abdominal CT, it was unlikely that tumor had heterogeneous components.

In our case, the expression of myogenin confirmed by immunohistochemistry and RT-PCR analysis led to the diagnosis of epiRMS. Twenty-four cases of epiRMS have been reported [2, 13–18]. In most cases, tumor cells showed diffuse and strong positive staining for desmin, which would be suggested a myogenic tumor. In our case, tumor cells showed only focal positive staining for desmin, which made it difficult for a correct diagnosis.

In our case, the PAX3/7-FKHR fusion genes were subjected to RT-PCR and karyotype-analysis.

The PAX3/7-FKHR fusion gene is specific to ARMS. The presence of the fusion gene was not confirmed in our case as in past epiRMS cases [2, 14, 16]. On cytogenetic (karyotypic) analysis, tumor cells showed highly complex karyotypes with triploidy and structural rearrangements. There was no description of cytogenetic analysis in the past epiRMS cases.

As Jo et al. described, our case showed diffuse sheet-like growth with abundant amphophilic cytoplasm and a clear large nucleus in the deep soft tissues of elderly patient and exhibited an aggressive clinical course [2]. The expression of myogenin confirmed by immunohistochemistry and RT-PCR analysis. In past cases, tumor cells showed diffuse and strong positive staining for desmin [2, 13–18]. In our case, however, tumor cells showed focal positive staining for desmin and was not comfirmed the expression of desmin by RT-PCR analysis. The reason there is no expression of desmin is unclear.

From the cytogenetical point of view, ARMS is an independent variant because of the recurrent translocations. ERMS, PRMS and SRMS exhibit complex karyotype with numeric and structural rearrangements. PRMS and SRMS occur mainly in elderly adults, while ERMS can also occur in elderly adults. Non-specific complex karyotypes with numeric and structural rearrangements may be common findings in adult RMS, apart from their morphological diversity. EpiRMS may also share a common finding in karyotypic analysis. Stock et al. argued that adult-type RMS is a single entity with wide morphological variety [7]. EpiRMS may also be one of the morphological diverse types in adult-type RMS.

In conclusion, a case of epiRMS occurring in an adult is reported. The differential diagnosis for epiRMS includes poorly differentiated carcinoma, malignant melanoma, and epithelioid sarcoma. It is difficult to distinguish epiRMS only by morphological analysis from other tumors that showed epithelioid morphology. Immunohistochemical and/or molecular analyses are needed to make the correct diagnosis. It is not clear whether epiRMS is an independent entity in RMS. Although it is difficult to properly treat adult RMS including epiRMS because of the aggressive clinical course, the correct diagnosis is needed for the discovery and improvement of future therapy. A larger-scale, multi-institute study is needed to provide more insight into epiRMS.

## Consent

Written informed consent was obtained from the next of kin of the patient for publication of this Case Report and any accompanying images.

## Additional file

Additional file 1: Figure S1. RT-PCR analysis. **Figure S2.** Karyotypic analysis of tumor cells. **Figure S3.** Additional immunohistochemical images. **Table S1.** Details of used antibodies. **Table S2.** The details of karyotype. **Table S3.** Primer sequences for the detection of Myo D1, Myogenin and PAX3/7-FKHR fusion gene.

## Abbreviations

RMS: Rhabdomyosarcoma
WHO: World Health Organization
ERMS: Embryonal rhabdomyosarcoma
ARMS: Alveolar rhabdomyosarcoma
PRMS: Pleomorphic rhabdomyosarcoma
SRMS: Spindle cell/sclerosing rhabdomyosarcoma
epiRMS: Epithelioid rhabdomyosarcoma
CT: Computed tomography
HU: Huns Hounsfield unit
RT-PCR: Reverse transcriptase polymerase chain reaction
