# Supplementary figures and images for: Epithelioid Rhabdomyosarcoma; a case report with immunohistochemical and molecular study
Source: Diagn Pathol. 2015 Jul 25;10:124. doi: 10.1186/s13000-015-0349-2 (PMC4514988; doi:10.1186/s13000-015-0349-2)

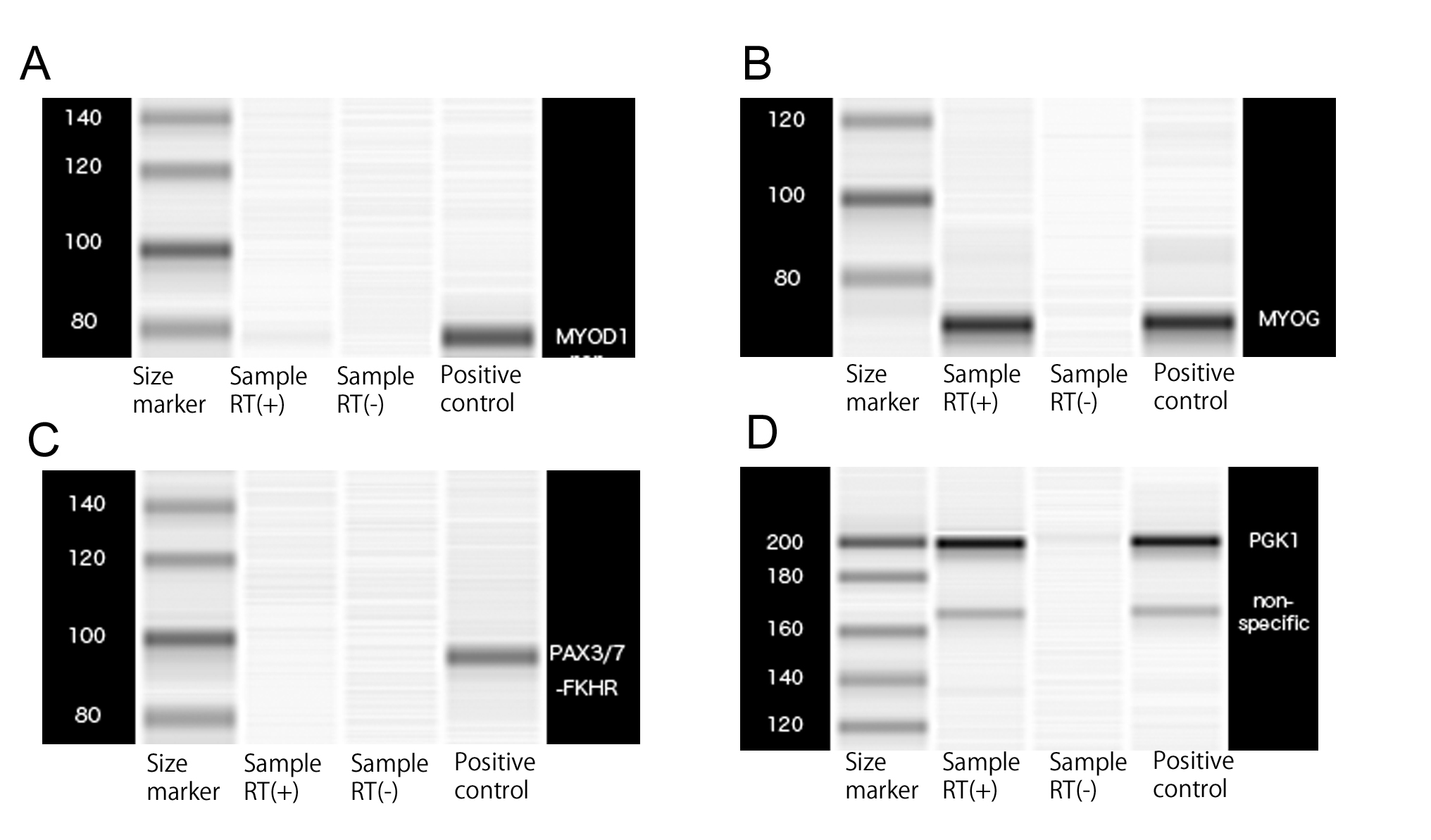

Supplement: Additional file 1: Figure S1. — RT-PCR analysis. Figure S2. Karyotypic analysis of tumor cells. Figure S3. Additional immunohistochemical images. Table S1. Details of used antibodies. Table S2. The details of karyotype. Table S3. Primer sequences for the detection of Myo D1, Myogenin and PAX3/7-FKHR fusion gene. [file 13000_2015_349_MOESM1_ESM.zip › Figure S1.jpg]

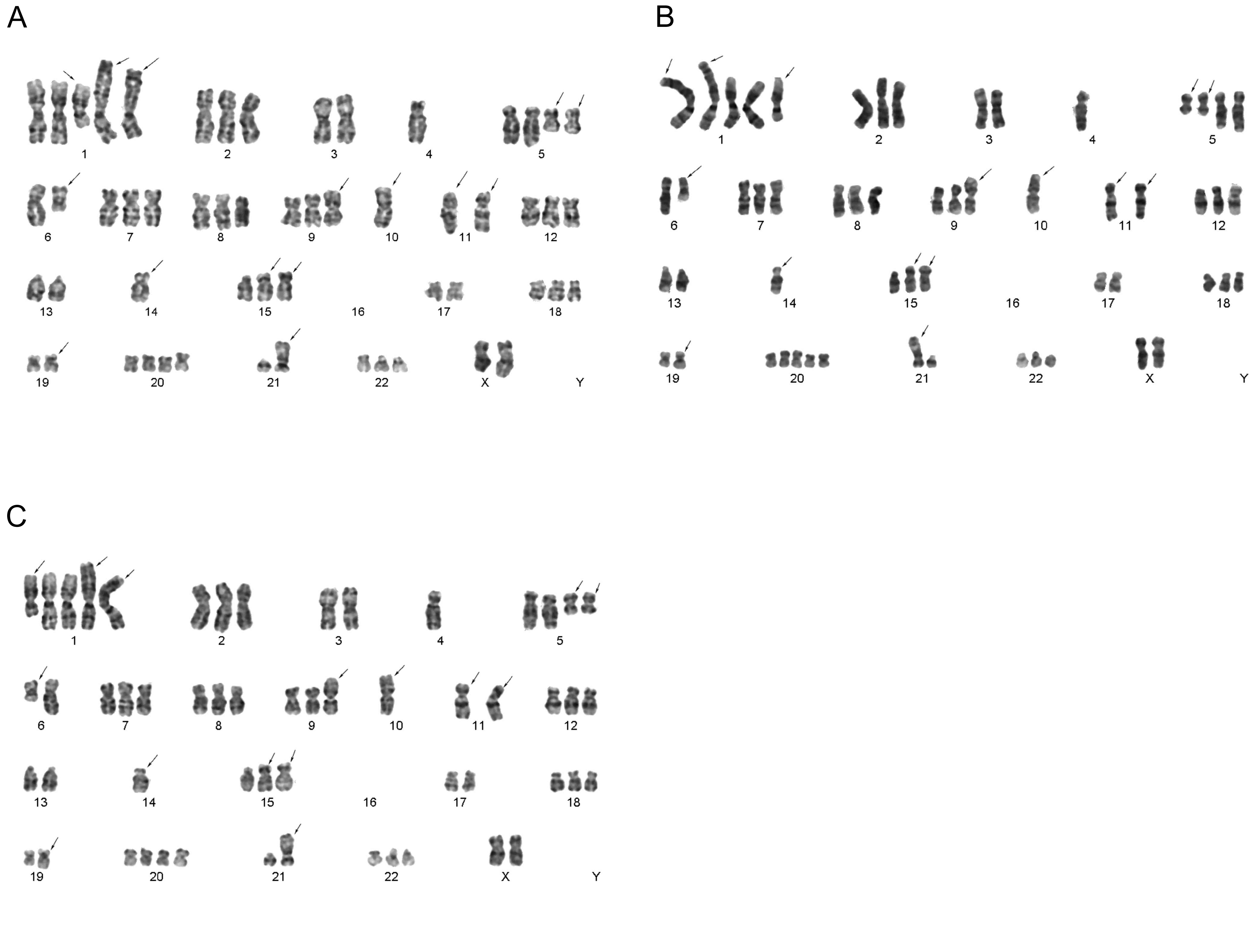

Supplement: Additional file 1: Figure S1. — RT-PCR analysis. Figure S2. Karyotypic analysis of tumor cells. Figure S3. Additional immunohistochemical images. Table S1. Details of used antibodies. Table S2. The details of karyotype. Table S3. Primer sequences for the detection of Myo D1, Myogenin and PAX3/7-FKHR fusion gene. [file 13000_2015_349_MOESM1_ESM.zip › Figure S2.jpg]

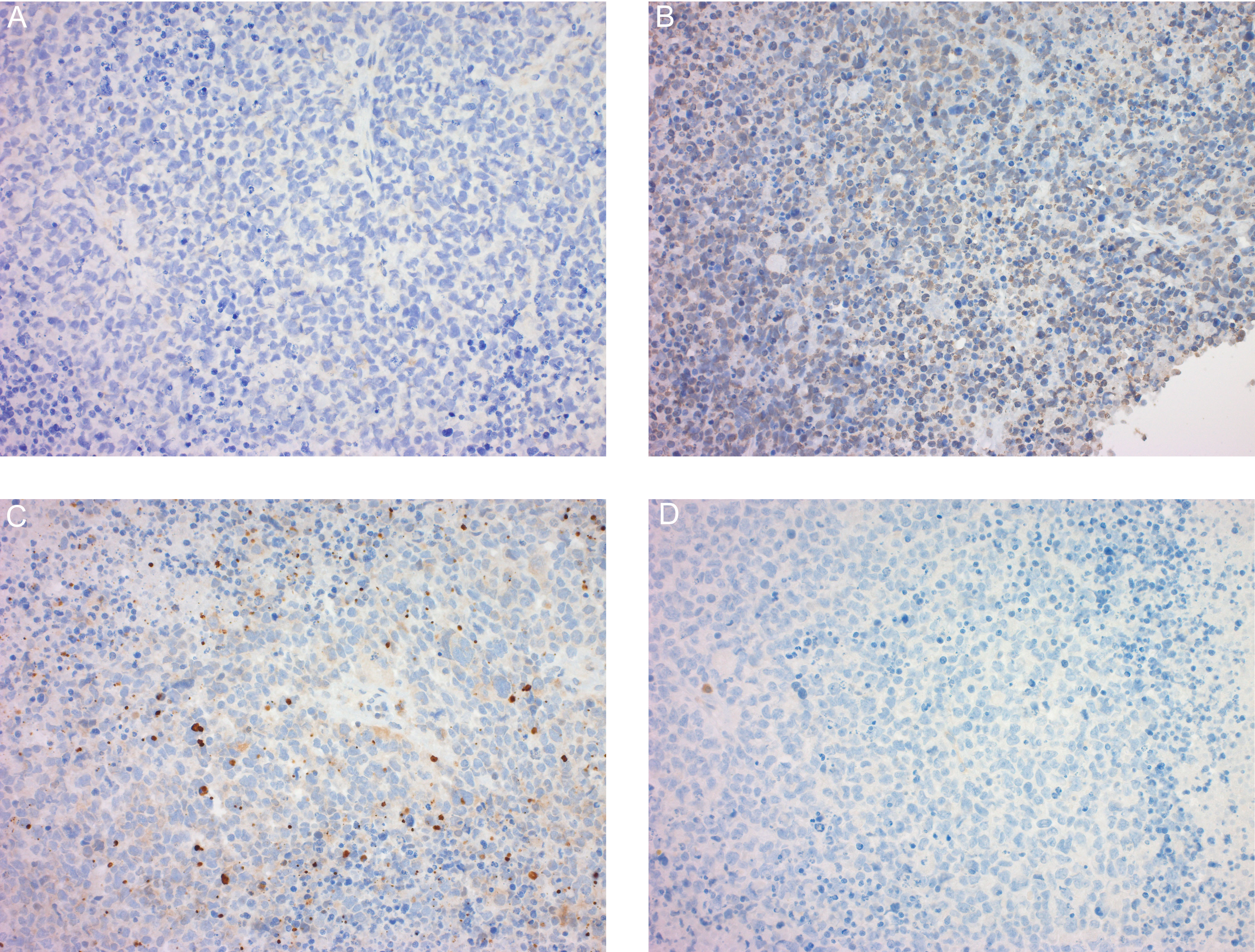

Supplement: Additional file 1: Figure S1. — RT-PCR analysis. Figure S2. Karyotypic analysis of tumor cells. Figure S3. Additional immunohistochemical images. Table S1. Details of used antibodies. Table S2. The details of karyotype. Table S3. Primer sequences for the detection of Myo D1, Myogenin and PAX3/7-FKHR fusion gene. [file 13000_2015_349_MOESM1_ESM.zip › Figure S3.jpg]
