# Supplementary material for: Epithelioid Rhabdomyosarcoma; a case report with immunohistochemical and molecular study
Source: Diagn Pathol. 2015 Jul 25;10:124. doi: 10.1186/s13000-015-0349-2 (PMC4514988; doi:10.1186/s13000-015-0349-2)
Supplement: Additional file 1: Figure S1. — RT-PCR analysis. Figure S2. Karyotypic analysis of tumor cells. Figure S3. Additional immunohistochemical images. Table S1. Details of used antibodies. Table S2. The details of karyotype. Table S3. Primer sequences for the detection of Myo D1, Myogenin and PAX3/7-FKHR fusion gene. [file 13000_2015_349_MOESM1_ESM.zip › renamed_79520.doc]

**Supplementary figure legends:**

Figure S1. RT-PCR analysis (A): Analysis of Myo D1 expression. (B): Analysis of Myogenin expression. (C): Analysis of PAX3/7-FKHR expression. (D): PKG1 RNA was amplified in this case sample and served as a control for amplifiable RNA. Alveolar Rhabdomyosarcoma was used as positive control in each assay.

Figure S2. Karyotypic analysis of tumor cells showed a highly complex karyotype, with triploidy and structural rearrangements. Three karyotype types were detected. (A,B,C) The multiple cytogenetic abnormalities are listed in Table S2.

Figure S3. Immunohistochemical images: Tumor cells stained negative for Sox10 (A), MDM2 (B), CDK4 (C), p16 (D).

**RNA Extraction and RT-PCR**

Tumor specimens from Formalin-Fixed Paraffin-Embedded (FFPE) block were deparaffinized by general technique and were lysed with proteinase K in appropriate lysis buffer. Total RNA was extracted from these lysates using RNAiso Blood (Total RNA Extraction Reagent, TaKaRa BIO, Otsu, Japan) following the manufacturer's recommendations. Obtained crude RNA was treated with DNaseI(Roche Applied Science, AG, Basel, Switzerland) and re-purified with RNeasy Mini Kit(Qiagen, Venlo, Netherlands) according to the manuals.

RT reaction was performed using ReverTra Ace qPCR RT Master Mix with gDNA Remover (TOYOBO CO., LTD. Life Science Department, Osaka, Japan), according to the manufacturer's protocol. RT products were diluted fivefold with TE (pH8.0) and used following PCR amplification.

PCR mixture of 10 uL volume contained 1uL diluted RT product, 5 pmol each of forward and reverse primers, 1xPCR buffer and 0.2 U of KOD FX neo (KOD DNA polymerase, TOYOBO). Primer sequences are summarized in Table S3.

PCR was performed using a T-100 Thermal Cycler (Bio-Rad Laboratories, Hercules, California, U.S.A.) with the following thermalcycling conditions: 94°C for 2 minutes, followed by 40 cycles of 98°C for 10 seconds and 61°C for 10 seconds. After the last cycle, samples were held at 15°C.

These amplified products were analyzed by microchip electrophoresis with Shimadzu MCE- 202 MultiNA (Shimadzu Corporation, Kyoto, Japan) and “gel-like” images were visualized from electropherograms with MultiNA Viewer software.

Table S1

Details of used antibodies

| **Antibody** | **Clone** | **Source** |
| --- | --- | --- |
| Cytokeratin | AE1/AE3 | DAKO |
| LCA | 2B11 + PD7/26 | DAKO |
| S-100 | polyclonal | Nichirei Biosciences |
| Sox10 | polyclonal | Cell Marque |
| Melan A | A103 | DAKO |
| Smooth muscle actin | 1A4 | DAKO |
| Desmin | D33 | DAKO |
| h-Caldesmon | h-CD | DAKO |
| MDM2 | 1B10 | Leica |
| CDK4 | polyclonal | Abcam |
| P16 | E6H4 | Roche |
| Myo D1 | 5.8A | DAKO |
| Myogenin | F5D | DAKO |
| Vimentin | V9 | Leica Biosystems |
| BAF-47 | 25/BAF47 | BD Transdnction |

Table S2

Tumor cells showed highly complex karyotypes with triploidy and structural rearrangements.

| **Cell** | **Karyotype** | **Interpretation** |
| --- | --- | --- |
| **Cell : 1** | 66, XX, -X, +add(1)(p13)x2, add(1)(q21), -3, -4, -4, +5, add(5)(q11. 2)x2, -6, add(6)(q13), add(9)(p22), -10, -10, i(10)(q10), -11, add(11)(q23)x2, -13, -14, -14, add(14)(p11. 2), der(15;21)(q10;q10)x2, -16, -16, -16, -17, -19, add(19)(q13. 3), +20, +add(21)(p11.2), +10mar | Abnormal triploidy cells bearing a highly complex karyotype were detected in metaphase cells by conventional G-banding techniques. |
| **Cell : 2** | 66, XX, -X, +add(1)(p13)x2, add(1)(q21), -3, -4, -4, +5, add(5)(q11. 2)x2, -6, add(6)(q13), add(9)(p22), -10, -10, i(10)(q10), -11, add(11)(q23)x2, -13, -14, -14, add(14)(p11. 2), der(15;21)(q10;q10)x2, -16, -16, -16, -17, -19, add(19)(q13. 3), +20, +20, +add(21)(p11.2), +9mar |  |
| **Cell : 3** | 66, XX, -X, +add(1)(p13)x2, add(1)(q21), -3, -4, -4, +5, add(5)(q11. 2)x2, -6, add(6)(q13), add(9)(p22), -10, -10, i(10)(q10), -11, add(11)(q23)x2, -13, -14, -14, add(14)(p11. 2), der(15;21)(q10;q10)x2, -16, -16, -16, -17, -19, add(19)(q13. 3), +20, +add(21)(p11.2), +11mar |  |

Table S3

Primer sequences for the detection of Myo D1, Myogenin and PAX3/7-FKHR fusion gene and RT-PCR protocol

| name | size(bp) |  | sequence | Refference |
| --- | --- | --- | --- | --- |
| PGK1 | 189 | forward | 5'-CAGTTTGGAGCTCCTGGAAG |  |
|  |  | reverse | 5'-CTGGCACTGCATCTCTTGGCC |  |
|  |  |  |  |  |
| PAX3/7 | 89 | forward | 5'-CCCTCCAACCCCATGAACCC |  |
| FKHR |  | reverse | 5'-ATGAACTTGCTGTGTAGGGACAG |  |
|  |  |  |  |  |
| MYOG | 64 | forward | 5'-CACTCCCTCACCTCCATCGT |  |
|  |  | reverse | 5'-CATCTGGGAAGGCCACAGA |  |
|  |  |  |  |  |
| MYOD1 | 76 | forward | 5'-TGCCACAACGGACGACTTC |  |
|  |  | reverse | 5'-CGGGTCCAGGTCTTCGAA |  |

The primers for PAX3/7 share a common sequence of PAX3 and PAX7.

**Reference**

1. Jin L, Majerus J, Oliveira A, Inwards CY, Nascimento AG, Burgart LJ et al. Detection of fusion gene transcripts in fresh-frozen and formalin-fixed paraffin-embedded tissue sections of soft-tissue sarcomas after laser capture microdissection and rt-PCR. Diagn Mol Pathol. 2003;12(4):224-30.

2. Park KH, Franciosi S, Leavitt BR. Postnatal muscle modification by myogenic factors modulates neuropathology and survival in an ALS mouse model. Nat Commun. England2013. p. 2906.
